# Sinapisms

**Published:** 1851-05

**Authors:** 


SINAPISMS.

A series of experiments were recently made in France, by Trousseau and Pidoux, for the purpose of ascertaining the best means for applying mustard as a sinapism. The experiments revealed the singular fact that mustard mixed with water is a better combination than mustard and vinegar, or mustard and acetic acid. The experiments showed that the combination of French mustard with acetic acid made the rubefacient action almost inert, but the same result did not obtain when English mustard was used. We think the reason of this is obvious. There is more oil left in the flour of French mustard than in that of the English, the acetic acid combines with the oil, and is thus rendered inert; and the volatile principle is not set free.

The most reliable mustard we have ever found for sinapisms, was the Lexington mustard, during the life of its originator, Nathan Burrowes. The only kind we have ever seen equal to it, is manufactured in this city by Tannehill & McAlister. We never saw it fail, where there was a chance for a sinapism to succeed. We have not tried the relative advantages of a mixture of Tannehill's mustard with warm or cold water, vinegar, or acetic acid, but if any of our friends wish to be miniature martyrs to science, we will try the mustard poultices upon them, and note the effects. B.

